# Association of *IKZF1* SNPs in cold medicine-related Stevens–Johnson syndrome in Thailand

**DOI:** 10.1186/s13601-019-0300-9

**Published:** 2019-11-22

**Authors:** Patchima Chantaren, Passara Jongkhajornpong, Mayumi Ueta, Vilavun Puangsricharern, Kaevalin Lekhanont, Phattrawan Pisuchpen, Pinnita Prabhasawat, Kanya Suphapeetiporn, Shigeru Kinoshita

**Affiliations:** 1 Department of Ophthalmology, Faculty of Medicine, Chulalongkorn University and Excellence Center for Cornea and Limbal Stem Cell Transplantation, King Chulalongkorn Memorial Hospital, Thai Red Cross Society, Bangkok, Thailand; 2 Department of Ophthalmology, Faculty of Medicine, Ramathibodi Hospital, Mahidol University, Bangkok, Thailand; 3 Department of Frontier Medical Science and Technology for Ophthalmology, Kyoto Prefectural University of Medicine, 465 Kajiicho, Hirokoji, Kawaramachi, Kamigyoku, Kyoto, 602-0841 Japan; 4 Department of Ophthalmology, Faculty of Medicine, Siriraj Hospital, Mahidol University, Bangkok, Thailand; 5 Department of Pediatrics, Faculty of Medicine, Chulalongkorn University and Excellence Center for Medical Genetics, King Chulalongkorn Memorial Hospital, the Thai Red Cross Society, Bangkok, Thailand

**Keywords:** Stevens–Johnson syndrome, Cold medicine, Severe ocular complications, SNPs, *IKZF1*

## Abstract

**Purpose:**

Our meta-analysis of several ethnic groups (Japanese, Korean, Indian, Brazilian) revealed a significant genome-wide association between cold medicine-related SJS/TEN (CM-SJS/TEN) with severe ocular complications (SOC) and *IKZF1* SNPs, suggesting that *IKZF1* might be a potential marker for susceptibility to CM-SJS/TEN with SOC. In this study, we examined the association between CM-SJS/TEN with SOC and the *IKZF1* SNPs in the Thai population.

**Methods:**

57 CM-SJS/TEN with SOC and 171 control samples were collected at Chulalongkorn University and Mahidol University. Genomic DNA samples were genotyped for the *IKZF1* SNPs at Kyoto Prefectural University of Medicine in Japan using the TaqMan SNP genotyping assay.

**Results:**

The four SNPs previously reported to be associated with CM-SJS/TEN with SOC in the Japanese were examined in the Thai samples. Although the number of Thai cases (n = 57) was small, a significant association between CM-SJS/TEN with SOC and *IKZF1* SNPs which included rs4917014 (T vs G, OR = 2.9, p = 0.0012, Pc = 0.0049), rs4917129 (T vs C, OR = 2.8, p = 0.0026, Pc = 0.010) and rs10276619 (G vs A, OR = 1.8, p = 0.012, Pc = 0.048) was identified.

**Conclusion:**

In addition to the Japanese, Korean and Indian populations, Thai cases with CM-SJS/TEN and SOC were significantly associated with *IKZF1* SNPs. With our previous report of the critical role of *IKZF1* in mucocutaneous inflammation, these results suggest that *IKZF1* is important in the pathogenesis of CM-SJS/TEN with SOC.

To the editor

Stevens–Johnson syndrome (SJS) and its severe type, toxic epidermal necrolysis (TEN), are acute inflammatory vesiculobullous reactions of the skin and mucosa including the ocular surface, oral cavity, and genitals. Severe ocular complications (SOC) appear in about half of SJS/TEN patients diagnosed by dermatologists [1]. Cold medicines (CM), including multi-ingredient cold medications and non-steroid anti-inflammatory drugs (NSAIDs) were the main causative drugs of SJS/TEN with SOC [2]. In the acute stage, in addition to skin eruption and erosion, SJS/TEN with SOC patients manifest severe conjunctivitis with corneal and conjunctival erosion and pseudo-membranes. Despite healing of the skin lesions, in the chronic stage of SJS/TEN with SOC, ocular surface sequelae such as severe dry eye, symblepharon, trichiasis, scaring of palpebra conjunctiva, and conjunctival invasion into the cornea may persist [3]. While the reported annual incidence of SJS/TEN is very rare (only 1–6/10^6^ individuals), its mortality rate is high (3% for SJS and 27% for TEN) [4]. We previously reported that the *IKZF1* gene was strongly associated with CM-SJS/TEN with SOC in Japanese patients [5]. In addition, a meta-analysis of several ethnic groups (Japanese, Korean, Indian, Brazilian) revealed a significant genome-wide association between CM-SJS/TEN with SOC and *IKZF1*, suggesting that *IKZF1* might be a potential marker for susceptibility to CM-SJS/TEN with SOC [5]. In this study, we examined the association between Thai CM-SJS/TEN with SOC and the *IKZF1* SNPs, known to be associated with the Japanese CM-SJS/TEN with SOC.

The CM-SJS/TEN with SOC and control samples were collected at Chulalongkorn University (King Chulalongkorn Memorial Hospital) and Mahidol University (Ramathibodi Hospital and Siriraj Hospital). Genomic DNA samples were genotyped for the *IKZF1* SNPs at Kyoto Prefectural University of Medicine in Japan. The study was approved by the institutional review board of all institutes. Protocol explanation and obtaining written informed consent were done in all participants before starting experimental procedures. All experimental processes were complied with the principles set forth in the Helsinki declaration. The diagnostic criteria of SJS/TEN were based on a confirmed history of acute onset of high fever, skin eruption with at least two sites of serious mucocutaneous involvement including the oral mucosa and the ocular surface [5]. Thai healthy volunteers were used as controls. CM was defined as the drug that patients took for relieving cold symptoms including nonsteroidal anti-inflammatory drugs (NSAIDS), acetaminophen, and other multi-ingredient cold medications [6]. We previously reported that in Japanese, for SJS/TEN with SOC, acetaminophen was a main drug of CM; 48% of the patients have taken acetaminophen before developing SJS/TEN with SOC [6]. In Thailand, paracetamol (which is equal to acetaminophen) might be also important causative drug of CM, because 20 of 57 (35%) patients have taken paracetamol before developing it. The patients were classified as having SOC if the following manifestations were detected; severe conjunctivitis, pseudomembrane, and epithelial defect on the ocular surface in the acute stage and/or ocular sequelae such as dry eye, trichiasis, symblepharon, and conjunctival invasion into the cornea in the chronic stage [6].

Of 57 CM-SJS/TEN with SOC, 23 were male and 34 were female; their age ranged from 6 to 73 years [median 42.3 ± 15.6 (SD) years]. The age at SJS/TEN onset ranged from 2 to 54 years (median 24.8 ± 13.8 years). The controls were 85 males and 86 females; their median age was 39.5 ± 14.3 years. Some of the CM-SJS/TEN patients and some of the controls had been included in our earlier studies [7].

Subjects were obtained DNA extraction from whole peripheral blood using the PAX gene blood DNA kits (Qiagen, Hilden, Germany) or from saliva using Oragene DNA (Kyodou International, Kanagawa, Japan). The TaqMan SNP genotyping assay (Applied Biosystems, Foster City, CA) was used for the genotypes of the *IKZF1* gene as previously reported [5]. Chi squared test was applied to a two-by-two contingency table for the allele frequency and the dominant and recessive models.

The 4 SNPs previously reported to be associated with CM-SJS/TEN with SOC in the Japanese were examined in the Thai samples. Although the number of Thai cases (n = 57) was small, we again found a significant association between CM-SJS/TEN with SOC and 3 *IKZF1* SNPs which included rs4917014 (T vs G, OR = 2.9, p = 0.0012, Pc = 0.0049), rs4917129 (T vs C, OR = 2.8, p = 0.0026, Pc = 0.010) and rs10276619 (G vs A, OR = 1.8, p = 0.012, Pc = 0.048) (Table 1). Our previous results of meta-analysis in the Japanese, Korean, Indian and Brazilian showed the significant associations in 3 *IKZF1* SNPs [rs4917014 (T vs G, OR = 2, p = 8.5 × 10^−11^) (which is equal to (G (minor allele) vs T (major allele), OR = 0.5) in the previous paper), rs4917129 (T vs C, OR = 2, p = 8.0 × 10^−9^) (which is equal to (C (minor allele) vs T (major allele), OR = 0.5 in the previous paper)) and rs10276619 (G vs A, OR = 1.8, p = 4.3 × 10^−9^)] [5]. Present results in the Thai population are in concordance with our previous report.

**Table 1:** Woolf’s correction

rs number of IKZF1 SNP	Genotypes		Case (N = 57)	Control (N = 171)	Allele 1 vs 2	Genotype 11 vs 12 + 22	Genotype 11 + 12 vs 22
					p-value Corrected p-value OR (95%CI)	p-value Corrected p-value OR (95%CI)	p-value Corrected p-value OR (95%CI)
rs897693	11	T/T	36/57 (63.2%)	114/171 (66.7%)	0.69	0.63	1.00
	12	T/C	17/57 (29.8%)	45/171 (26.3%)	–	–	–
	22	C/C	4/57 (7%)	12/171 (7%)	–	–	–
rs4917014	11	T/T	46/57 (80.7%)	100/171 (58.5%)	*1.22.E−03*	*2.46.E−03*	0.0619
	12	G/T	11/57 (19.3%)	61/171 (35.7%)	*4.89.E−03*	*9.86.E−03*	–
	22	G/G	0/57 (0%)	10/171 (5.8%)	*2.91* (1.49–5.68)	*2.97* (1.44–6.13)	–
rs4917129	11	T/T	47/57 (82.5%)	109/171 (63.7%)	*2.59.E−03*	*8.48.E−03*	*4.97.E−02*
	12	T/C	10/57 (17.5%)	51/171 (29.8%)	*1.03.E−02*	*3.39.E−02*	0.199
	22	C/C	0/57 (0%)	11/171 (6.4%)	*2.82* (1.40–5.68)	*2.67* (1.26–5.66)	8.24* (0.48–142.08)
rs10276619	11	G/G	25/57 (43.9%)	52/171 (30.4%)	*1.21.E−02*	0.063	*2.97.E−02*
	12	G/A	26/57 (45.6%)	78/171 (45.6%)	*4.84.E−02*	–	0.119
	22	A/A	6/57 (10.5%)	41/171 (24%)	*1.76* (1.13–2.74)	–	2.68 (1.07–6.70)

Our functional analysis of SNPs of the *IKZF1* gene revealed that the ratio of the splicing isoforms Ik2/Ik1 could be affected by *IKZF1* SNPs significantly associated with susceptibility to CM-SJS/TEN with SOC [5]. The quantity of the Ik2 isoform is increased in disease-protective genotypes of *IKZF1* (rs4917014 G/G and rs10276619 A/A) [5]. As Ikaros 2, an Ik2 isoform lacks the DNA-binding ability and seems to be dominant-negative. It is possible that the function of Ikaros, the protein of *IKZF1*, is enhanced in CM-SJS/TEN with SOC [5].

Ikaros is a transcription factor that regulates numerous biological events. It was reported that Ikaros-null mice lack B-lineage cells, NK cells, peripheral lymph node- and fetal T-cells, thus Ikaros family members regulate important cell-fate decisions in the development of the adaptive immune system [8]. On the other hand, we have reported that epithelium might be contribute to the pathobiology of CM-SJS/TEN with SOC [9]. Thus, we produced K5-Ikzf1-EGFP transgenic mice (Ikzf1 Tg) by introducing the Ik1 isoform into cells expressing keratin 5, which is expressed in epithelial tissues such as the epidermis and conjunctiva and found that mucocutaneous inflammation was exacerbated in Ikzf1-Tg mice. They developed dermatitis with some having blepharoconjunctivitis [10]. Histological analysis showed not only dermatitis but also tissue inflammation in the blepharoconjunctiva, tongue, and paronychia [10], similar to the findings in patients in the acute state of SJS/TEN with SOC [9]. Our studies demonstrated that *IKZF1* could play a critical role in maintaining mucocutaneous homeostasis [10] and suggested that it might be implicated in the aggravation of mucocutaneous inflammation seen in the presence of CM-SJS/TEN with SOC [10].

In addition to the Japanese, Korean and Indian in our previous report [5], CM-SJS/TEN with SOC was significantly associated with *IKZF1* SNPs in the Thai cases. With our previous report of the critical role of *IKZF1* in mucocutaneous inflammation [8], these results suggest that *IKZF1* is important in the pathogenesis of CM-SJS/TEN with SOC.
